# Organization and metamorphic remodeling of the nervous system in juveniles of *Phoronopsis harmeri* (Phoronida): insights into evolution of the bilaterian nervous system

**DOI:** 10.1186/1742-9994-11-35

**Published:** 2014-04-28

**Authors:** Elena N Temereva, Eugeni B Tsitrin

**Affiliations:** 1 Department of Invertebrate Zoology, Biological faculty, Lomonosov State University, Leninskie Gory 1/12, Moscow 119992, Russian Federation; 2 Institute of Developmental Biology, Russian Academy of Sciences, Moscow 117808, Russia

**Keywords:** Phylogeny, Nervous system, Evolution, Lophophorata, Metamorphosis, Deuterostomia, Protostomia, The last common bilaterian ancestor

## Abstract

**Background:**

Metamorphic remodeling of the nervous system and its organization in juvenile may shed light on early steps of evolution and can be used as an important criterion for establishing the relationships among large groups of animals. The protostomian affiliation of phoronids does not still have certain morphological and embryological proofs. In addition, the relationship of phoronids and other former “lophophorates” is still uncertain. The resolving of these conflicts requires detailed information from poorly investigated members of phoronids, such as *Phoronopsis harmeri*.

**Results:**

During metamorphosis, the juvenile consumes the nerve elements of the larval hood. Two dorsolateral groups of larval perikarya remain and give rise to the dorsal ganglion, which appears as the “commissural brain”. The juvenile inherits the main and minor tentacular nerve rings from the larva. Although the larval tentacles are directly inherited by the juvenile in *P. harmeri*, the ultrastructure and location of the definitive tentacular neurite bundles change greatly. Innervation of the juvenile lophophore exhibits a regular alternation of the intertentacular and abfrontal neurite bundles. The giant nerve fiber appears at early stage of metamorphosis and passes from the right group of dorsolateral perikarya to the left side of the body.

**Discussion:**

The metamorphic remodeling of the phoronid nervous system occurs in two different ways: with complete or incomplete destruction of organ systems. The morphology of the lophophore seems similar to those of the former members of “Lophophorata”, but its innervation differs greatly. These findings support the separation of bryozoans from Lophophorata and establish a need for new data on the organization of the brachiopod nervous system. The nervous system of the phoronid juvenile is organized as an epidermal nerve plexus but exhibits a nerve center in the anterior portion of the body. The simultaneous presence of both the apical organ and anlage of the cerebral ganglion in phoronids at the larval stage, and the reduction of the apical organ during metamorphosis support the Trochea theory and allow to suggest the presence of two nervous centers in the last common ancestor of the Bilateria. Phoronids retained some plesiomorphic traits and can be regarded as one of the most primitive groups of lophotrochozoans.

## Introduction

Phoronids are marine benthic animals with biphasic life cycles. Most of phoronids have a planktotrophic larva, which lives in plankton for several months [1,2] and then undergoes catastrophic metamorphosis. Detailed metamorphosis studies can help clarify some stages of early evolution [3,4]. Because metamorphosis recapitulate a number of phylogenetic events, its study is valuable to define the relationships and phylogenetic positions between taxa that do not currently occupy a clear phylogenetic position among other bilateria.

The phylum Phoronida has been classified into the protostomian clade through molecular phylogenetic analyses [5,6]. However, phoronid morphology and embryology have more in common with deuterostomes than protostomes [7,8]. For this reason, the affiliation of phoronids with the protostomian clade cannot be regarded as strictly established.

A lack of data on phoronid development remains a critical barrier to placing these organism in the tree of life. The organization and development of the nervous system are traditionally used for comparative analysis. In phoronids, the development and organization of the larval nervous system exhibits deuterostome-like features [8,9]. At the same time, we do not have sufficient data regarding the metamorphic remodeling and organization of the nervous system in the juvenile. The fate of the larval nervous system during metamorphosis and the organization of the juvenile nervous system are unknown. This knowledge may provide insight into phoronid phylogeny and may help establish relationships between phoronids and other Bilateria.

Phoronid metamorphosis has been studied many times by light microscopy [10-14]. The remodeling of nervous system has never been studied by a combination of immunocytochemistry, confocal laser scanning microscopy, and transmission electron microscopy. Some metamorphic stages of *Phoronis pallida* have been described in only one paper [15]. According to that brief description, the juvenile nervous system develops before metamorphosis and is integrated into that of the larva. Unfortunately, the fate of each element of the larval nervous system was not traced, and the origin of the dorsal ganglion, which is the main element of definitive nervous system, is still unclear.

Traditionally, phoronids, brachiopods, and bryozoans were all merged into a superphylum group, Lophophorata. This integration was based on a morphological peculiarity common between all lophophorates: the presence of lophophore – a special outgrowth of mesosome bearing tentacles that surround the mouth. The first molecular analysis data revealed that the phylogenetic group “Lophophorata” does not exist and that the Bryozoa form a separate stem within Lophotrochozoa [5]. These data were confirmed by subsequent results that demonstrated that Bryozoa belonged among Polyzoa [16]. According to recent results [17,18], phoronids and brachiopods are closely related and together form a united clade called Brachiozoa. However, phylogenetic analyses have suggested a close relationship between Bryozoa and Brachiopoda and have refuted the existence of Brachiozoa [19]. Interestingly, according to early data [20], phoronids were once combined with bryozoans into the group Podaxonia because they both have common patterns of metamorphosis, including enormous growth of the ventral side. The most recent phylogenomic data support the monophyly of Lophophorata and reveal the presence of an Ectoproct-Phoronid clade [21]. Taken together all these data indicate that the relationships between the former members of “Lophophorata” are still uncertain. A comparative analysis of the innervation of a structure as specific as the lophophore may help to clarify the “Lophophorata” phylogeny.

The primary aim of this study is to comprehensively describe the fate of the nervous system during metamorphosis and its organization in juveniles of *Phoronopsis harmeri*.

## Results

### Description of the competent larva

The organization of *P. harmeri* competent larva, which is ready for metamorphosis, was given in several previous papers [8,10,22]. The serotonin-like, FMRFamide-like, and alpha-tubulin-like immunoreactivity has been investigated in the larval nervous system of *P. harmeri* in a recent publication [8]. Here, we briefly describe the overall larval morphology and organization of the nervous system in competent larva of *P. harmeri* (Table 1).

**Table 1:** The nerve elements of phoronid larvae and their fate during metamorphosis

**Name of nerve element (labelled in Figure**1**C)**	**Expression of 5HT**	**Expression of FMRFamide**	**Expression of alfa-tubulin**	**Fate during metamorphosis and (immunoreactivity in juveniles)**
apical organ (ao)	+	+	+	completely lost
median neurite bundle of the preoral lobe (mn)	+	+	+	completely lost
anterior marginal neurite bundle of the preoral lobe (am)	+	―	+	completely lost
ventrolateral branches (vlb)	+	―	+	completely lost
posterior marginal neurite bundle of the preoral lobe (pm)	+	+	+	completely lost
sensory field (sf)	+	―	―	completely lost
neurites and perikarya innervating the oral field (ofn) Can be observed by TEM only	―	―	―	completely lost
tentacular nerve ring (=main nerve ring) (tn)	+	+	+	completely maintained (5HT, FMRFamide, alfa-tubulin)
minor nerve ring (mn)	―	―	+	completely maintained (alfa-tubulin)
mediofrontal tentacular neurite bundle (mf)	―	+	+	undergoes changes: can not be detected via LSCM and changes in number of neurites (from 100 to 10) (alfa-tubulin)
laterofrontal tentacular neurite bundles (lf)	―	―	+	completely lost; definitive neurite bundles form *de novo* (alfa-tubulin)
medioabfrontal tentacular neurite bundle	―	―	―	appears in juveniles (alfa-tubulin)
lateroabfrontal neurite bundles (la)	+	―	+	completely maintained (5HT, alfa-tubulin)
two dorsolateral groups of perikarya (gp)	+	―	―	completely maintained and give rise to the definitive dorsal ganglion (5HT)
telotroch nerve ring (ttn)	+	―	―	completely lost
neurites and nerve cells innervating the epidermis of the larval trunk (sg(=mdn?)) (neurite bundles of the second group [8])	+	―	―	probably maintained and give rise to the most dorsal neurites (5HT)
neurites of the esophagus (es)	+	+	+	completely maintained (5HT, FMRFamide, alfa-tubulin)
neurites and perikarya innervating the midgut (pmg)	―	+	―	completely maintained (FMRFamide, alfa-tubulin)
anal nerve ring (ar)	―	+	―	probably undergoes changes, because in juveniles exhibits 5HT-like immunoreactivity
neurites and perikarya innervating the metasomal sac (nms)	+	+	―	completely maintained (5HT, FMRFamide, alfa-tubulin)

" + " indicates the presence of the feature; "―" indicates its absence.

The larval body is divided into three regions: the hood (preoral lobe) with the apical organ and the preoral ciliated band, the collar region with the oral field and tentacles, which bear postoral ciliated band, and the trunk with telotroch (Additional file 1).

**Figure 1:**
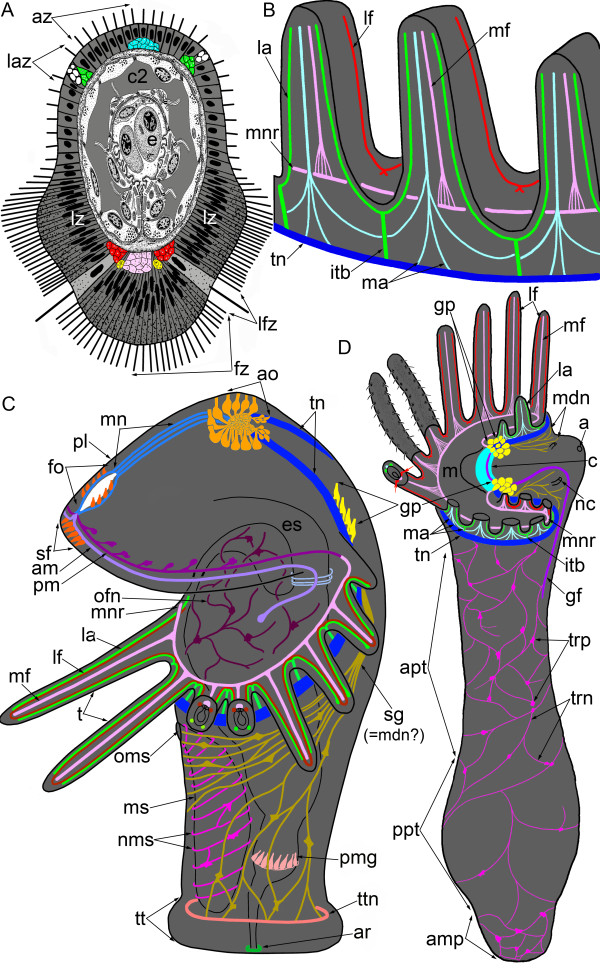
**Schemes of organization of the nerve system in juvenile (A, B, D) and competent larvae (C) of *Phoronopsis harmeri*.** Nerve elements are shown by different colors. Although the homology between larval and definitive nerve elements is still uncertain, some nerve elements, which juvenile certainly inherits from larva, are indicated by the same colors in juvenile and in larva. **(A)** The scheme of cross section of tentacle. **(B)** The scheme of innervation of definitive tentacles, which are viewed from the abfrontal side. **(C)** The nervous system of competent larva, which is ready for metamorphosis. All nerve elements, which were observed by TEM and immunocytochemical staining, are shown. Larva is viewed from the left; the apical is at the top, the ventral side is to the left. Picture was changed from Temereva and Tsitrin [8]; abbreviation are shown in the Table 1 and in Figure 4[8]. **(D)** Scheme of whole nervous system in 10-day-old juvenile. The lophophore is simplified; tentacles of the left side are removed. The oral side is to the left. The number of nerve fibers along the body reflects the density of the nerve plexus in different body parts. Abbreviations: a – anus; amp – ampulla; apt – anterior portion of the body; az – abfrontal zone; c – commissure; c2 – mesocoel; e – erythrocyte; fz – frontal zone; gf – giant fiber; gp – group of perikarya; itb – intertentacular branch; la – lateroabfrontal neurites; laz – lateroabfrontal zone; lf – laterofrontal neurites; lfz – laterofrontal zone; lz – lateral zone; m – mouth; ma – medioabfrontal neurites; mdn – most dorsal neurites, which probably correspond to second group of larval neurites (sg); mf – mediofrontal neurites; mnr – minor nerve ring; nc – nephridial channel; pt – posterior portion of the body; tn – tentacular (main) nerve ring; trn – trunk neurites; trp – trunk perikarya.

The nervous system in competent larvae of *P. harmeri* consists of the following elements: an apical organ, a median neurite bundle, an anterior and posterior marginal neurite bundles, a frontal organ, a sensory field, a tentacular neurite bundle (main nerve ring), two dorsolateral groups of perikarya, a minor nerve ring, five neurite bundles in each tentacle, a telotroch nerve ring, an anal nerve ring, and nerve cells innervating the epidermis of the trunk, the esophagus, metasomal sac, and the midgut [8] (Figure 1C).

### Serotonin-like immunoreactivity in the metamorphic nervous system

**Figure 2:**
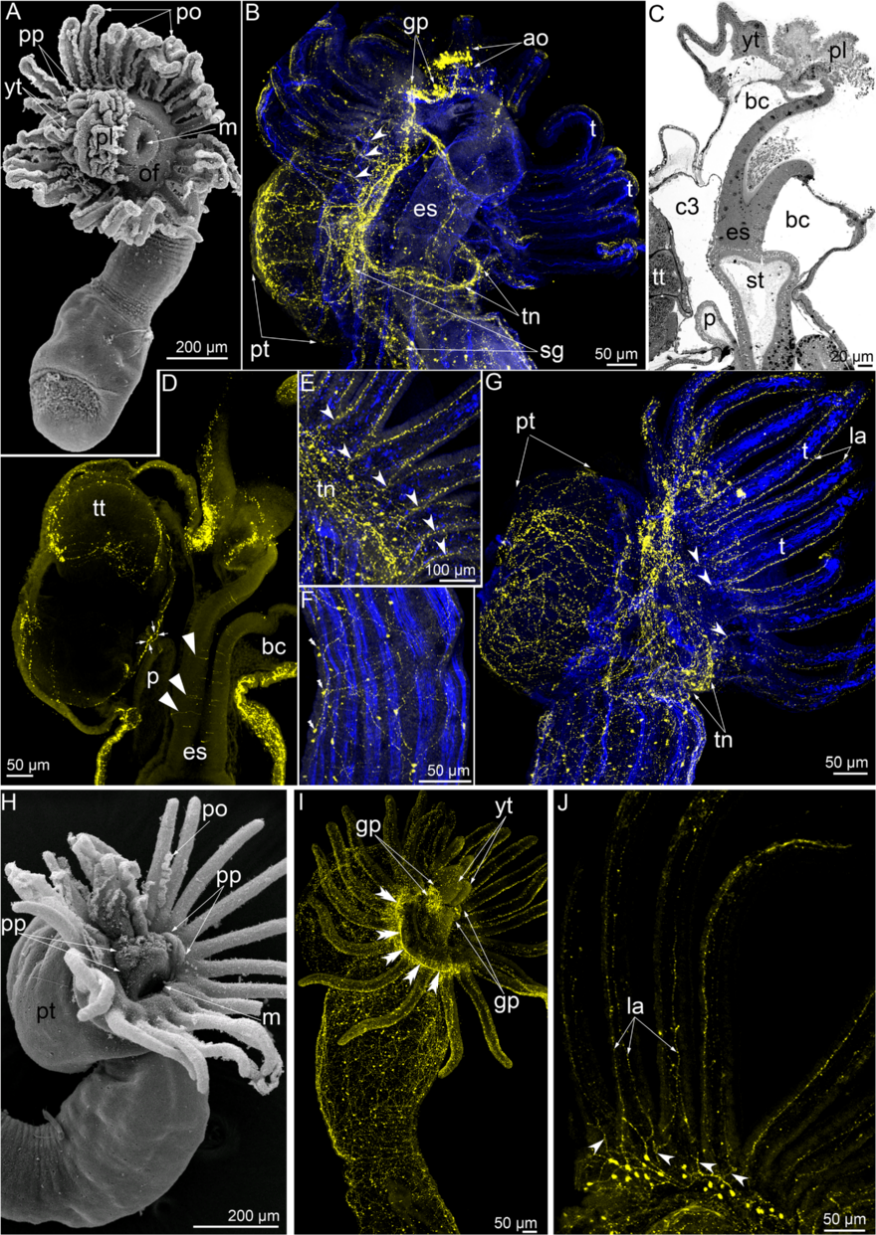
**Serotonin-like immunoreactive nervous system in *Phoronopsis harmeri *during the first stages of metamorphosis. (A-G)** Animals at one of the first stages of metamorphosis (the “hood-eating stage”). **(H-J)** Animals at the stage when the postoral ciliated band is ingested. In all images, the apical is at the top, and the oral side is to the right. Z-projections **(B, D, E, G, H)** of animals after mono- and double staining for serotonin (yellow) and phalloidin (blue). **(A)** Whole animal viewed from the top; SEM. **(B)** Anterior portion of the body. The intertentacular branches are indicated by opened arrowheads. **(C)** Sagittal semithin section of the anterior portion of the body. **(D)** Optical sagittal section of the anterior part of the body. The serotonin-like immunoreactive cells in the esophagus are indicated by closed arrowheads. The nerve ring around the anus is indicated by arrows. **(E)** The portion of tentacular (main) nerve ring with intertentacular branches (arrowheads). **(F)** Middle part of the body with nerve plexus and nonsensory perikarya (double close arrowheads). **(G)** Whole anterior part of the body. **(H)** Anterior part of the body; SEM. **(I)** Whole anterior part of the animal. The main nerve ring is indicated by double arrowheads. **(J)** Part of the main nerve ring and lateroabfrontal neurites. The intertentacular branches are indicated by open arrowheads. Abbreviations: ao – apical organ; bc – blastocoel; c3 – trunk coelom; es – esophagus; gp – groups of perikarya; m – mouth; la – lateroabfrontal neurite bundles; of – oral field; p – proctodaeum; pl – preoral lobe; po – postoral ciliated band; pp – remnant of the hood; pt – posterior part of the larval body with the telotroch; sg – neurites of the second group; st – stomach; t – tentacle; tn – tentacular (main) nerve ring; tt – telotroch; yt – youngest tentacles.

Metamorphosis begins with the eversion of the metasomal sac (Additional file 1). Immediately after eversion, the hood epidermis begins to macerate (Figure 2A). Then, the marginal part of the hood together with the sensory field, marginal nerves, the frontal organ, and the median nerve are engulfed by the mouth and consumed by the juvenile. The central part of the hood including the apical organ can be observed during the first stages of metamorphosis (Figure 2B). The apical organ maintains connection with the tentacular nerve ring. Two dorsolateral groups of perikarya are associated with the main tentacular nerve ring and are situated behind the apical organ at the bases of youngest tentacles (Figure 2B). The main tentacular nerve ring forms serotonin-like immunoreactive intertentacular branches, which bifurcate and penetrate into adjacent tentacles (Figure 2B).

Neurites of the second group (=neurites and nerve cells innervating the epidermis of the larval trunk), which are prominent in competent larva and spread along the lateral sides of the trunk (see [8]), are retained and pass from the tentacular nerve ring to the proximal end of the everted metasomal sac (Figure 2B). Larval trunk neurites and perikarya form a net around the remnant of larval posterior body part and the telotroch.

The hood is engulfed gradually, and the apical organ is consumed by the juvenile (Figure 2C). The major tentacular nerve ring maintains its continuity and consists of numerous neurites, which are parallel to each other and associated with perikarya that are scattered among them (Figure 2E, G). The thin serotonin-like immunoreactive intertentacular branches are inherited from larva and still visible (Figure 2E, G). In each tentacle, two lateral serotonin-like immunoreactive neurites remain (Figure 2E, G). The innervations of the larval body remnant remains and is provided with numerous neurites and perikarya (Figure 2D, G). The telotroch is surrounded by numerous thin neurites, which also form the ring around the anus (Figure 2D, G). In median optical sections, serotonin-like immunoreactive cells are evident in the esophageal epithelium (Figure 2D). The body of juvenile is covered by net of neurites and perikarya, which mostly do not contact the epidermis surface and are apparently nonsensory (Figure 2F).

At later stages, the hood is completely engulfed except for two dorsolateral parts (Figure 2H). These hood remnants are located near the youngest tentacles, on both sides of the epistome anlage, which arises from the dorsal portion of the esophagus (Figure 2H). At stage when the postoral ciliated band is ingested, the main tentacular nerve ring is prominent and begins with two dorsolateral aggregations of perikarya, which are located in the hood remnants (Figure 2I). The innervation of tentacles can be observed with higher magnification (Figure 2J). Serotonin-like immunoreactive intertentacular branches are still evident. Two lateroabfrontal serotonin-like immunoreactive neurites remain in each tentacle (Figure 2J).

**Figure 3:**
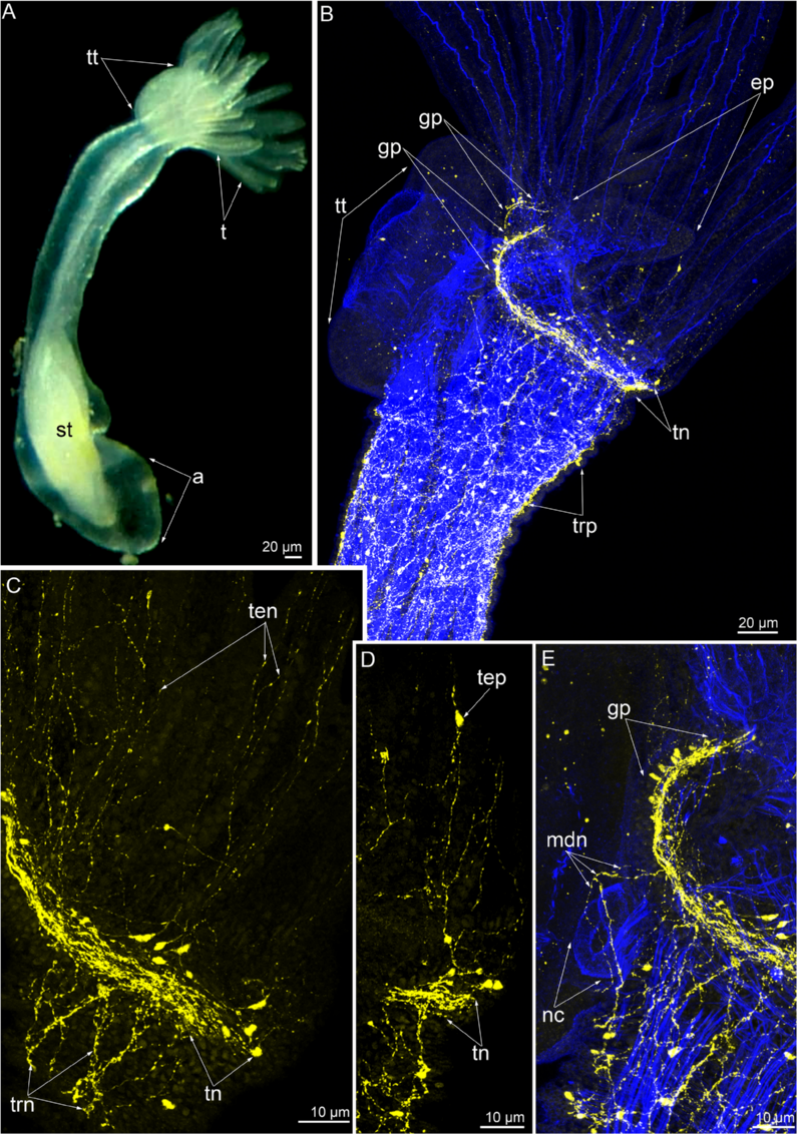
**Serotonin-like immunoreactive nervous system in 3-day-old *Phoronopsis harmeri*.** In all images, the apical is at the top, and the oral side is to the right. Z-projections **(B-E)** of animals after mono- and double staining for serotonin (yellow) and phalloidin (blue). **(A)** Live whole animal. **(B)** Anterior portion of the body viewed from the right side. **(C)** A part of the main nerve ring with multipolar perikarya, which give rise to the tentacular neurites. **(D)** Neurites and immunoreactive perikarya in tentacles. **(E)** The most dorsal portion of the main nerve ring with group of dorsolateral perikarya and the most dorsal neurites around the nephridial channel. Abbreviations: ep – epistome; gp – dorsolateral group of perikarya; mdn – most dorsal neurites; nc – nephridial channel; st – stomach; t – tentacle; ten – tentacular neurites; tep – perikarya in the tentacles; tn – tentacular (main) nerve ring; trn – neurites innervating the trunk; trp – perikarya innervating the trunk; tt – telotroch.

In the 3-day-old juvenile, the larval trunk becomes very short, and the telotroch becomes smaller (Figure 3A). The neurites and perikarya are absent in the larval trunk remnant (the epidermis of the aboral side of the anterior body part) (Figure 3B). There are several neurites, which spread between the main nerve ring and nephridial channels (Figure 3E). These neurites are probably remnants of the neurites of the second group, which are numerous in larvae. The epidermis of the rest of the body contains numerous multipolar perikarya, which usually do not contact the surface of the epidermis (Figure 3B). The main nerve ring is the most prominent element of the juvenile nervous system. Several groups of multipolar perikarya are located above the tentacular nerve ring on the lateral and oral body sides (Figure 3C). These perikarya form long, thin neurites, which penetrate into the tentacles. The location of serotonin-like immunoreactive neurites in tentacles does not follow a consistent pattern. Solitary multipolar perikarya are situated in the tentacles (Figure 3D). Two dorsolateral groups of perikarya are still visible at this stage (Figure 3E). These perikarya contact the surface of the epidermis and do not form neurites that extend into the tentacles.

**Figure 4:**
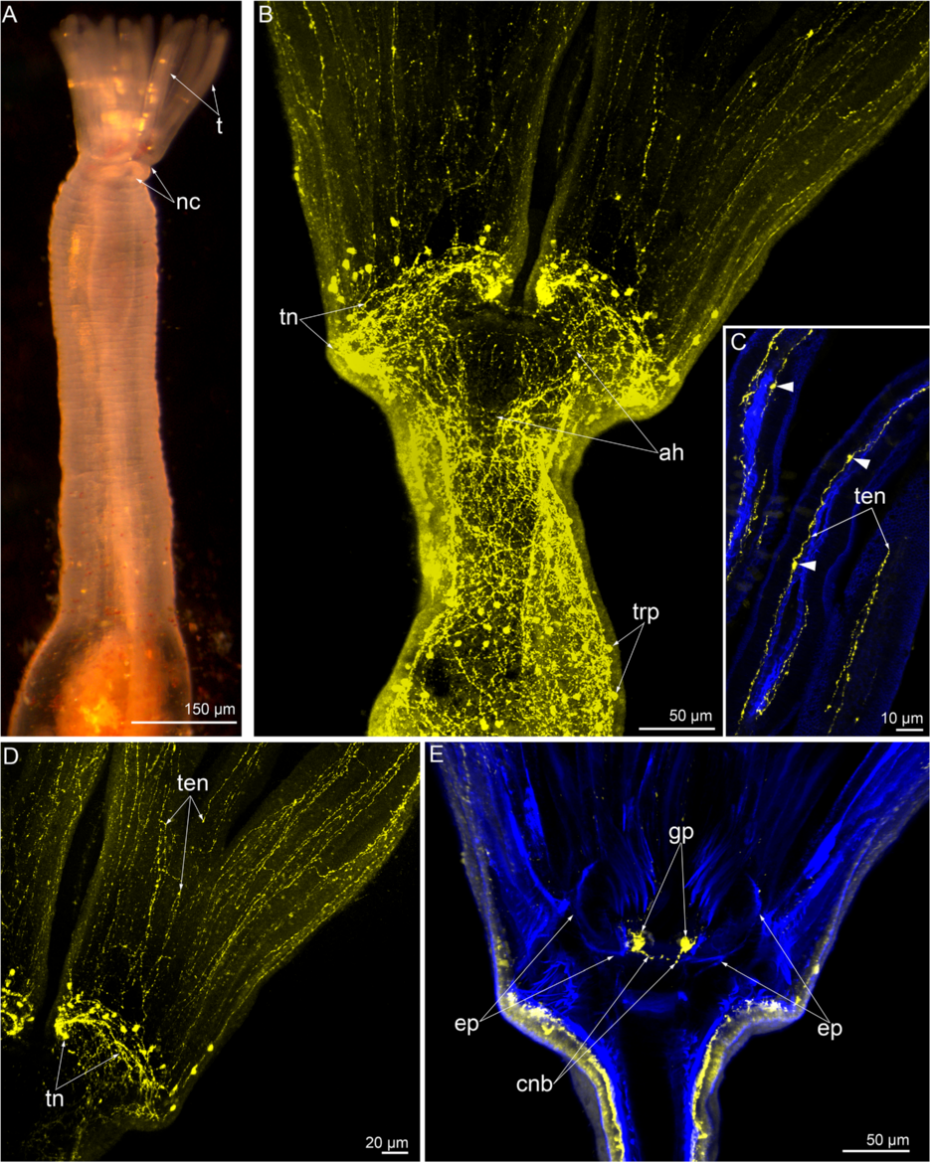
**Serotonin-like immunoreactive nervous system in 10-day-old *Phoronopsis harmeri*.** In all images, the apical is at the top. Z-projections **(B-D)** of animals after mono- and double staining for serotonin (yellow) and phalloidin (blue). **(A)** Live animal; anterior part of the body. The anal side is to the right. **(B)** Anterior portion of the body viewed from the anal side. **(C)** Optical sections of the several tentacles with tentacular neurites and solitary perikarya (arrowheads). **(D)** The right half of the lophophore with the main nerve ring and tentacular neurites. **(E)** Optical frontal section of the anterior body part viewed from the oral side. Abbreviations: ah – area of the anal hill; cnb – central neurite bundles; ep – epistome; gp – dorsolateral group of perikarya; nc – nephridial channel; t – tentacle; ten – tentacular neurites; tn – tentacular (main) nerve ring; trp – perikarya innerveting the trunk.

By the ninth day after metamorphosis has begun, the telotroch disappears completely, and the 10-day-old juvenile looks like an adult (Figure 4A). The epidermis of the body contains numerous serotonin-like immunoreactive perikarya, which are mostly multipolar, and neurites, which form a thick net around the body (Figure 4B). The anal hill, where the telotroch was located until the 9-day-old stage, lacks neurites and perikarya (Figure 4B). The main nerve ring consists of several transversal neurites and multipolar perikarya, which give rise to the tentacle neurites (Figure 4D). Some perikarya are located above the tentacular nerve ring, and solitary perikarya occur in the tentacles (Figure 4C). Two dorsolateral groups of serotonin-like immunoreactive perikarya remain in the 10-day-old animal. These groups appear as large, bright aggregations, which are located in the epistome base near the youngest tentacles (Figure 4E). Two thick neurite bundles pass from the dorsolateral aggregation of perikarya towards the center of the epistome.

### FMRFamide-like immunoreactive nervous system

**Figure 5:**
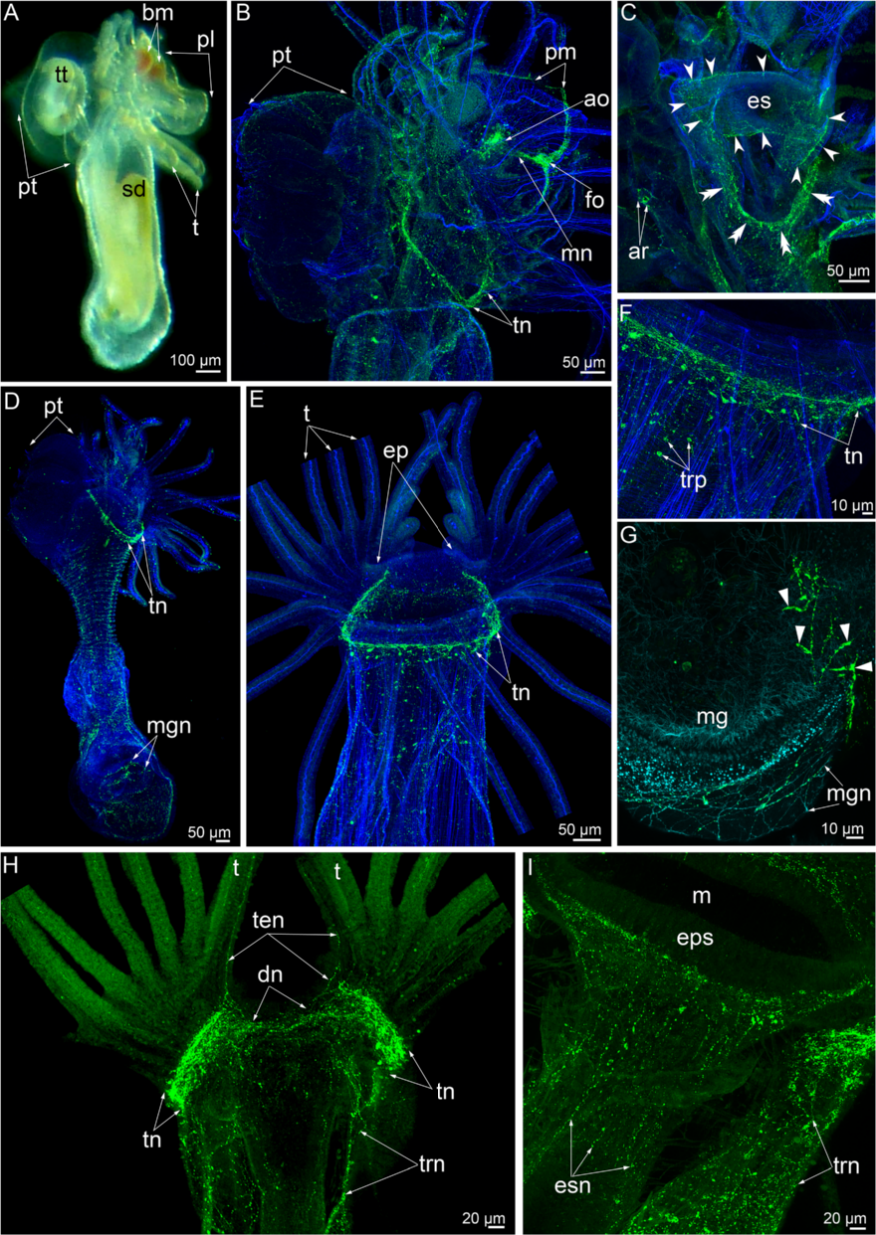
**FMRFamide-like immunoreactive nervous system in *Phoronopsis harmeri *at consecutive stages of metamorphosis.** In all images, the apical is at the top. Z-projections **(B-I)** of animals after mono- and double staining for FMRFamide (green), phalloidin (blue), and alpha-tubulin (cyan). **(A)** Live whole animal at first stage of metamorphosis. The oral side is to the right. **(B)** The same stage; anterior portion of the body and the preoral lobe. The oral side is to the right. **(C)** The same stage; anterior part of the body with esophageal and anal nerve elements. The esophageal immunoreactive neurites and perikarya are indicated by open arrowheads. The tentacular nerve ring is indicated by double arrowheads. **(D)** The stage when the postoral ciliated band is ingested. Whole animal viewed from the right side. **(E)** A 3-day-old juvenile viewed from the oral side; anterior part of the body. **(F)** The same stage; a part of the tentacular nerve ring. **(G)** The same stage; the part of the midgut with neurites and perikarya (closed arrowheads). **(H)** A 3-day-old juvenile viewed from the anal side. **(I)** The same stage; the upper part of the esophagus. Abbreviations: ar – anal nerve ring; ao – apical organ;dn – dorsal neurites; bm – blood masses; ep – epistome; eps – epithelium of the esophagus; es – esophagus; esn – neurites innervating the esophagus; fo – frontal organ; m – mouth; mg – midgut; mgn – neurites in the midgut; mn – medial neurite bundle; pl – preoral lobe; pm – posterior marginal neurite bundle; pt – posterior part of the larval body with the telotroch; sd – stomach diverticulum; t – tentacle; ten – tentacular neurites; tn – tentacular (main) nerve ring; trn – neurites innervating the trunk; trp – perikarya innervating the trunk; tt – telotroch.

During the first stages of metamorphosis (Figure 5A), all of the larval FMRFamide-like immunoreactive elements (as described previously [8]), including the esophageal neurites and circumanal ring, are visible (Figure 5B, C). With time, the main nerve ring remains whereas most of the other elements of the larval nervous system disappear (Figure 5D). Fifteen minutes after metamorphosis has begun, FMRFamide-like immunoreactive neurites in tentacles cannot be found. All nervous elements of the larval hood are engulfed. The The tentacular (main) nerve ring is evident at all stages of metamorphosis. In the 3-day-old juvenile, it is the main element of the FMRFamide-like immunoreactive nervous system (Figure 5E, F). The main nerve ring consists of neurites, which are mostly circular, and multipolar perikarya (Figure 5F). The main nerve ring is horseshoe-like in shape, and its two branches pass to the dorsal side of the epistome (Figure 5E). In the epistome base, the two branches of the “horseshoe” connect: the group of thin neurites passes across the epistome base (Figure 5H). With higher magnification, these thin neurites cross between the two branches of the main nerve ring and the thin neurites in the youngest tentacles are evident (Figure 5H).

FMRFamide-like immunoreactive neurites and perikarya of inner organs remain and can be traced at all stages during metamorphosis (Figure 5B-D, G, I). Numerous perikarya are located in the epithelium of the midgut (Figure 5D, G). These perikarya have a narrow apical part and a wide basal part with several basal processes, which form a net around the midgut. FMRFamide-like immunoreactive neurites are found along the esophagus. They are mostly orientated longitudinally and form a thick net around the esophagus (Figure 5I). A few FMRFamide-like immunoreactive perikarya are scattered along the juvenile body. They are multipolar, and their projections form a net around the body (Figure 5E, F).

### alpha-tubulin-like immunoreactive elements

**Figure 6:**
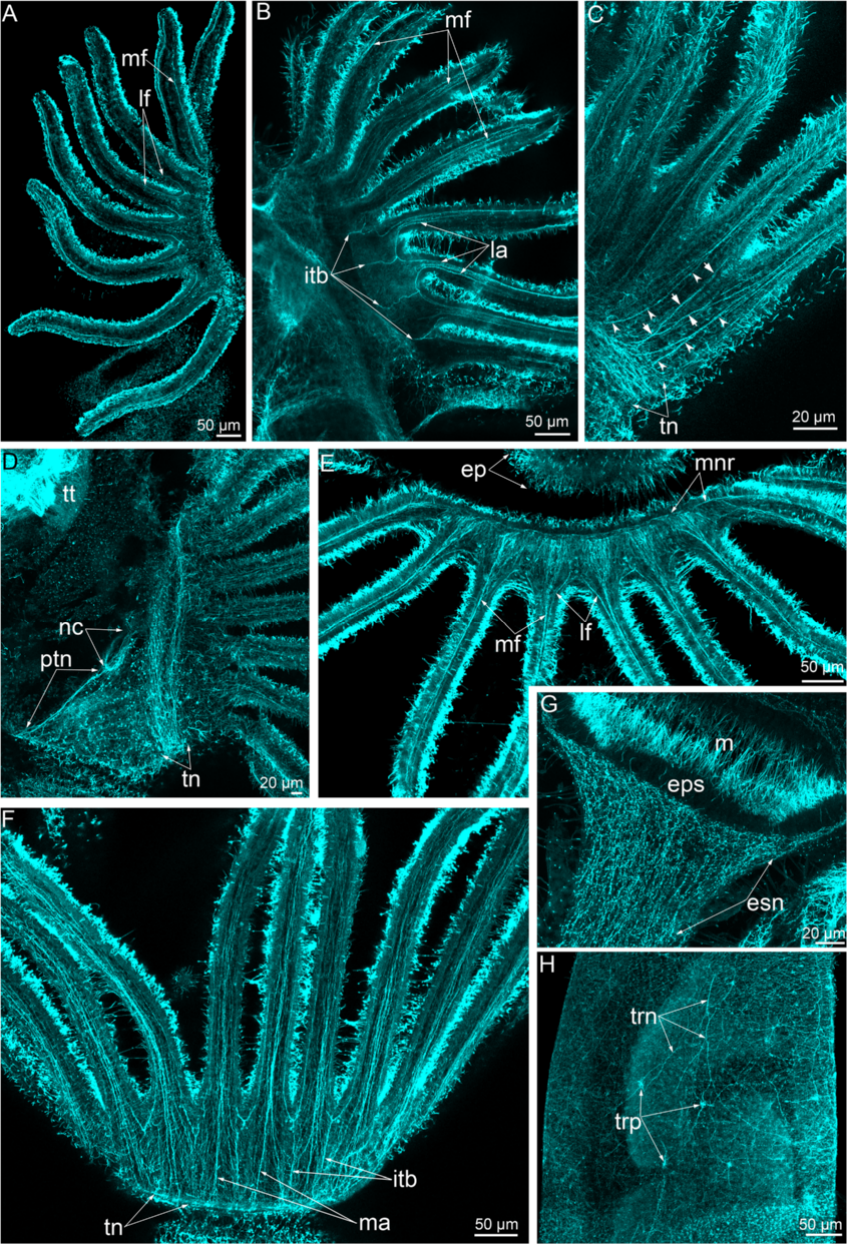
**Innervation of the lophophore and alpha-tubulin immunoreactive elements in *Phoronopsis harmeri *at different stages of metamorphosis.** In all images, the apical is at the top. **(A)** First stage of metamorphosis; several tentacles viewed from the frontal side. The oral side is to the right. **(B)** The same stage; a part of the lophophore with tentacles viewed from the abfrontal side. The oral side is to the right. **(C)** The stage when the postoral ciliated band is ingested, several tentacles viewed from the abfrontal side. Medioabfrontal neurite bundles are indicated by arrowheads. Intertentacular branches are indicated by arrows. The oral side is to the right. **(D)** The same stage; anterior part of the body viewed from the right. **(E)** A 3-day-old juvenile; a part of the lophophore with tentacles viewed from the frontal side. **(F)** The same stage; a part of the lophophore with tentacles viewed from the abfrontal side. **(G)** A 3-day-old juvenile; the upper part of the esophagus. **(H)** A 3-day-old juvenile; a part of the trunk body wall. Abbreviations: ep – epistome; eps – epithelium of the esophagus; itb – intertentacular branch; la – lateroabfrontal neurites in tentacle; lf – laterofrontal neurites in tentacle; m – mouth; ma – medioabfrontal neurites in tentacle; mf – mediofrontal neurites in tentacle; mnr – minor nerve ring; nc – nephridial channel; ptn – prominent trunk neurite bundle; tn – tentacular (main) nerve ring; trn – trunk neurites; trp – trunk perikarya; tt – telotroch.

During the first stages of metamorphosis, tentacles retain the larval pattern of innervation (as previously shown [8]) with one mediofrontal, two laterofrontal, and two lateroabfrontal neurite bundles (Figure 6A, B). Moreover, the lateroabfrontal neurite bundles originate from the intertentacular branches, which are also evident in competent larva (Figure 6B). The connection between the frontal neurite bundle and the minor nerve ring is not evident.

At later stages of metamorphosis, the pattern of tentacles innervations differs from that of the larva because the prominent medioabfrontal neurite bundle appears in each tentacle (Figure 6C). The intertentacular neurite bundles remain and form two branches, which penetrate into the adjacent tentacle (Figure 6C). At this stage, the prominent trunk neurite is located under the channel of the left nephridium and probably corresponds to the giant nerve fiber (Figure 6D).

In the 3-day-old juvenile, the staining against alpha-tubulin reveals a complex scheme of tentacle innervations. The frontal median neurite bundle passes along the frontal side of each tentacle (Figure 6E). It begins with a large aggregation of many thin neurites, which originate from the thin minor nerve ring. The laterofrontal neurites are retained, but in some cases their connections between the tentacles are not evident (Figure 6E). Regular groups of neurite bundles occur on the abfrontal side of the lophophore (Figure 6F). The neurite of one group passes from the main nerve ring to the base between tentacles: this is the intertentacular branch. Along each intertentacular branch, several left and right neurites originate (Figures 1B, 6F). The terminal part of each intertentacular branch bifurcates and forms two neurites, which penetrate into adjacent tentacles. Thus, each tentacle contains two lateral neurites, which originate from two intertentacular branches. The abfrontal neurite bundle originates from two main neurites, which intertwine with neurites that originate from the intertentacular branch (Figure 6F). As a result, intertentacular and abfrontal branches alternate along the main nerve ring.

The thick net of neurites around the esophagus can be observed by staining of alpha-tubulin. This net is mostly formed by longitudinal neurites (Figure 6G). The net of neurites and some perikarya along the trunk of the juvenile is revealed by alpha-tubulin staining (Figure 6H).

### Changes in the Phoronid nervous system at the ultrastructural level

We traced the ultrastructural changes of phoronid nerve elements at several crucial stages of metamorphosis. In this study, we described the organization of the main nerve elements at these stages.

**Figure 7:**
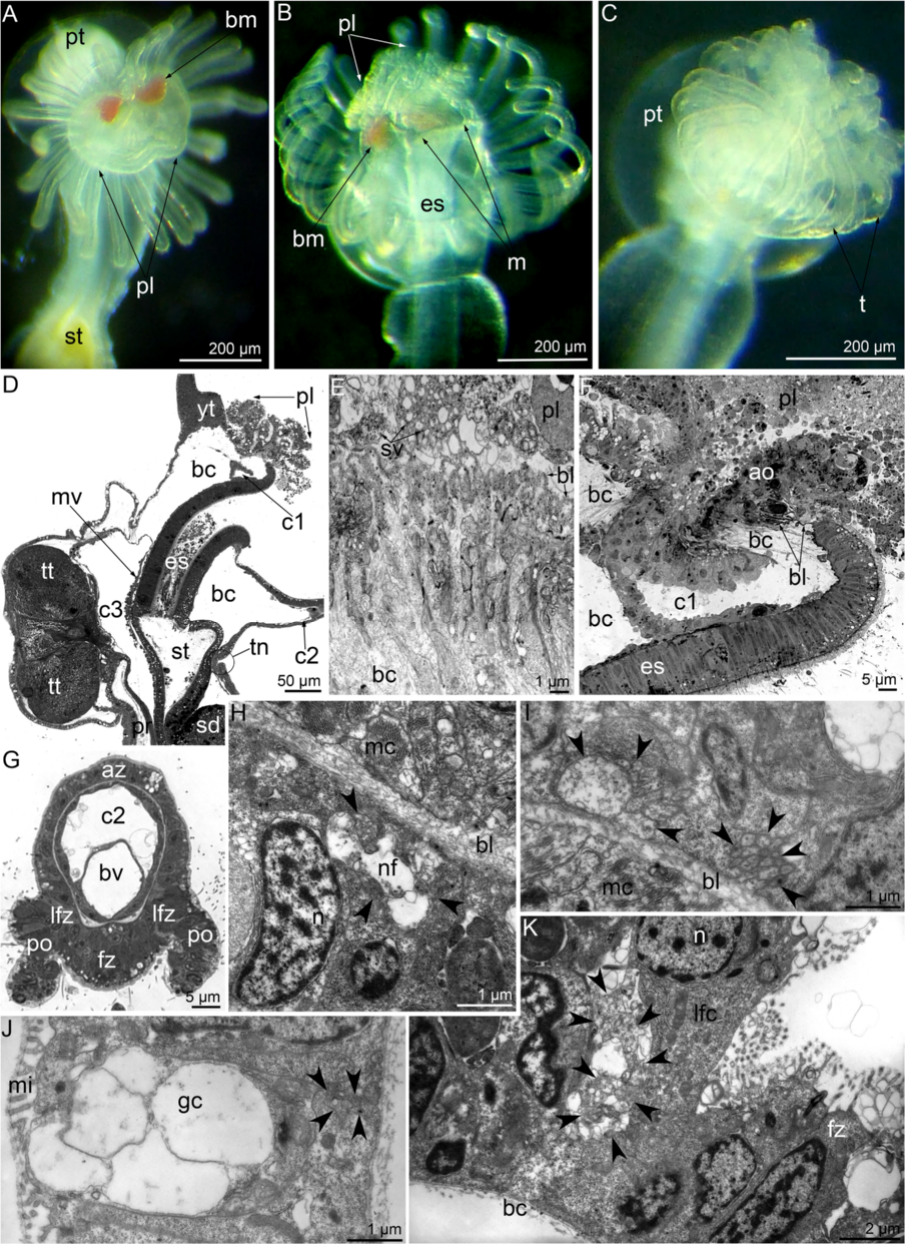
**Morphological and ultrasrtuctural changes of the larval hood and tentacles at the firsts stages of metamorphosis of *Phoronopsis harmeri. *(A-C)** The anterior portion of the body of live animals. **(D, F, G)** Semithin sections. **(E, H-K)** Thin section. **(A)** First stage of metamorphosis: the hood (pl) remains its integrity. **(B)** The next (second) stage of metamorphosis: the hood (pl) turns into cellular debris and engulfed. **(C)** The third stage of metamorphosis: larval tentacles (t) form a “cup” and surround the hood. **(D)** The second stage of metamorphosis, sagittal section; the oral side is to the right, the anal side is to the left, the apical is at the top. (**E)** Thick basal lamina (bl) and spacious blastocoel (bc) under degenerated cells of the preoral lobe and the apical organ, which still remains synaptic vesicles (sv). **(F)** Sagittal section of the protocoel (c1), degenerated hood (pl), and the apical organ (ao). **(G)** The cross section of the tentacle, which starts to acquire the definitive style via the peeling of the postoral ciliated band (po). **(H)** The mediofrontal neurite bundle (arrowheads). **(I)** The medioabfrontal neurite bundles (arrowheads). **(J)** The lateroabfrontal neurite bundle (arrowheads), which is associated with gland cell (gc). **(K)** Laterofrontal neurite bundle (arrowheads) is associated with laterofrontal sensory cell (lfc), which undergoes the cell death. Abbreviations: az – abfrontal zone; bm – blood masses; bv – blood vessel; c2 – tentacular coelom; c3 – trunk coelom; es – esophagus; fz – frontal zone; lfz – laterofrontal zone; m – mouth; mc – muscle cell; mi – microvilli; mv – median blood vessel; n – nucleus; nf – nerve fiber; pt – posterior part of the larval body with the telotroch; sd – stomach diverticulum; st – stomach; tn – main nerve ring; tt – telotroch.

Immediately after the eversion of the metasomal sac, the preoral lobe maintains its physical integrity (Figure 7A), but it undergoes cell death shortly thereafter. Five minutes after the beginning of metamorphosis, the preoral lobe degenerates into an aggregation of cellular debris (Figure 7B, D). The larval apical organ also disintegrates into cellular debris (Figure 7F). The macerated epithelium of the hood and the apical organ are consumed by juvenile whose bending tentacles form a closed “cup” (Figure 7C). When the epidermis and the apical organ are peeled off, the body remains covered only by the thick basal lamina (Figure 7E, F). Above the basal lamina, degenerated neurites with synaptic vesicles are found (Figure 7E). The basal lamina is associated with thick longitudinal bundles of collagenous fibers, which extend to the upper wall of the protocoel inherited from the larva (Figure 7E, F). The disintegration of the postoral ciliated band occurs simultaneously with the consumption of the hood. The epithelium of the postoral ciliated band, including the sensory laterofrontal cells, is squeezed out of the epidermis (Figure 7G) and forms a continuous cellular rope, which is subsequently consumed. The tentacular neurite bundles, which are present in the larva, can be found within the first few minutes of the juvenile, but their ultrastructure changes greatly. In the juvenile, the mediofrontal neurite bundle consists of 15 neurites, whereas in the larva, it consists of 80 to 100 neurites [8]. Some neurites have a large diameter and electron-lucent cytoplasm that contains dense core synaptic vesicles (Figure 7H). The laterofrontal neurite bundles lose contact with the basal lamina and undergo degeneration (Figure 7K). The medioabfrontal neurite bundles are not found in larval tentacles [8] but appear in juveniles as two bundles, each of which consists of 10 to 20 neurites. These neurites contain electron-lucent synaptic vesicles (Figure 7I). The lateroabfrontal neurite bundles do not change in comparison with larvae. In the juvenile, they consist of 7 to 10 neurites, which are associated with gland cells (Figure 7J).

**Figure 8:**
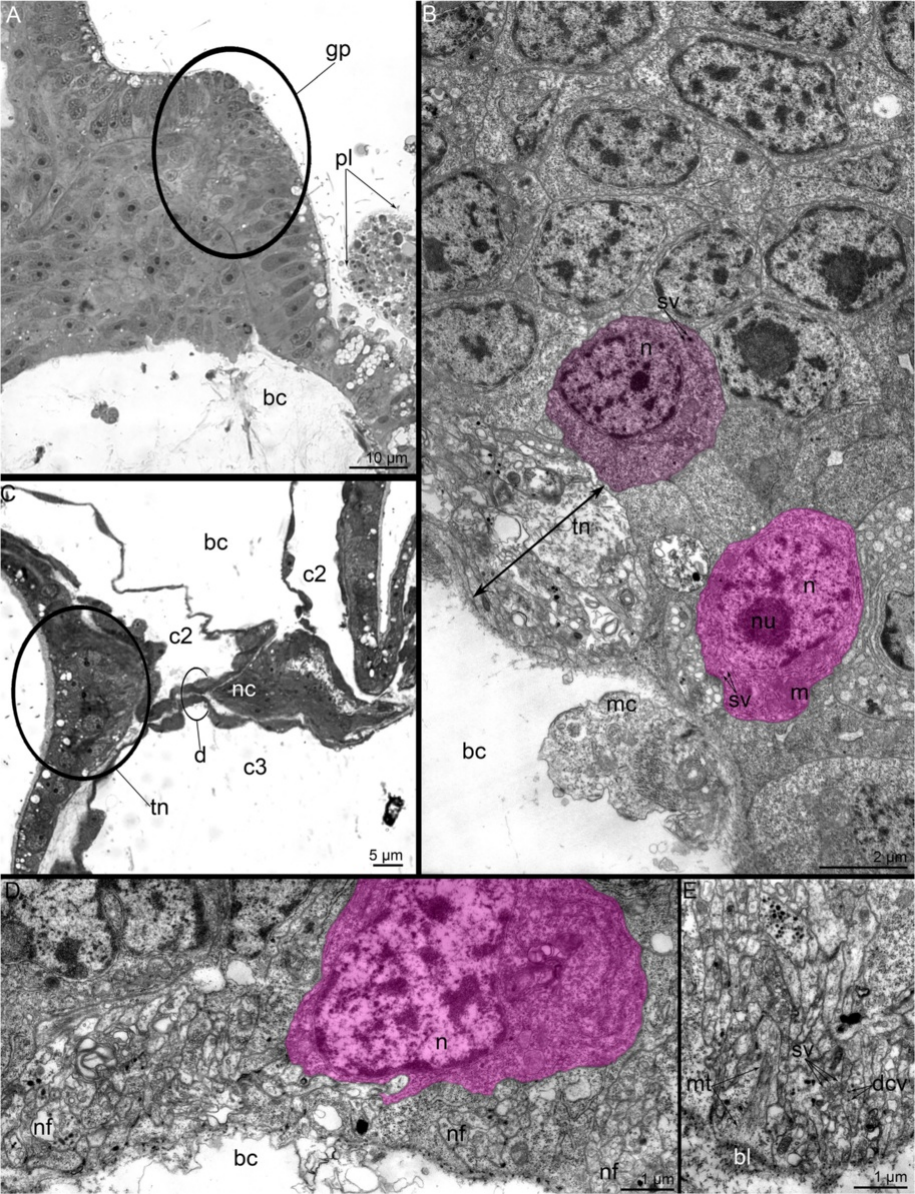
**Organization of main nerve elements at first stages of metamorphosis of *Phoronopsis harmeri.*** Sagittal semithin **(A, C)** and thin **(B, D-E)** sections. **(A)** One of dorsolateral groups of perikarya. **(B)** Perikarya (pink) of dorsolateral group, which is associated with the main nerve ring. **(C)** The main nerve ring. **(D)** Perikaryon (pink) in the main nerve ring. **(E)** The neuropil of the main nerve ring. Abbreviations: bc – blastocoel; c2 – tentacular coelom; c3 – trunk coelom; d – diaphragm; dcv – dense-core vesicle; gp – groups of perikarya; m – mitochondrion; mc – muscle cell; mt – microtubule; n – nucleus; nc – nephridial channel; nf – nerve fiber; pl – preoral lobe; sv – synaptic vesicle; tn – main nerve ring.

The metamorphic animal maintains the dorsolateral groups of perikarya. The number of these perikarya increases during metamorphosis. These are two groups of roundish cells that bear a large nucleus with a nucleolus. They have an electron-dense cytoplasm that contains a few synaptic vesicles, mitochondria, and rough endoplasmic reticulum (Figure 8A, B). These perikarya connect with the tentacular neurite bundle, which does not change during metamorphosis. The tentacular neurite bundle is located on the border between the mesocoel and the metacoel (Figure 8C). As in the larva, the tentacular neurite bundle in the juvenile consists of numerous neurites, which are associated with large perikarya (Figure 8D, E).

**Figure 9:**
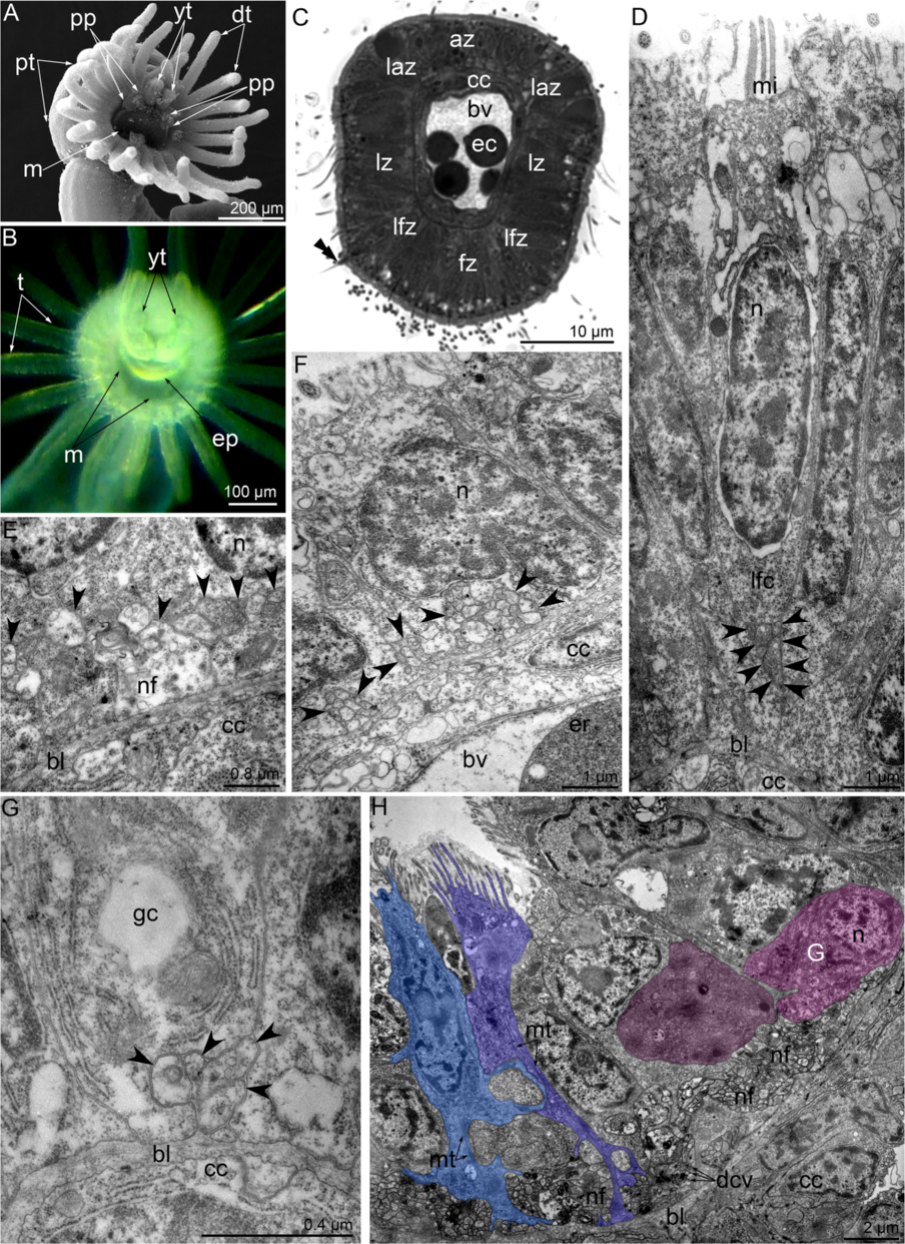
**The latest stages of metamorphosis of *Phoronopsis harmeri*. (A)** Metamorphic animal, which underwent all catastrophic changes in 30 minutes after metamorphosis start. **(B-H)** A 3-day-old juvenile. **(A)** The head region of metamorphic animal viewed from the oral side; SEM. **(B)** The head region of live juvenile viewed from the oral side. **(C)** The cross section of the tentacle: new postoral ciliated band is formed (the long microvilly of laterofrontal cell are shown by double arrowheads). **(D)** The laterofrontal cell, which is associated with laterofrontal neurite bundle (arrowheads). **(E)** The mediofrontal neurite bundle (arrowheads). **(F)** The medioabrfontal neurite bundles (arrowheads). **(G)** The lateroabfrontal neurite bundle (arrowheads), which is associated with gland cell. **(H)** The main nerve ring: sensory (blue) and nonsensory (pink) perikarya. Abbreviations: az – abfrontal zone; bl – basal lamina; bv – blood vessel; cc – coelomic lining; dcv – dense-core vesicle; dt – definitive tentacle; ec – erythrocyte; ep – epistome; fz – frontal zone; G – Golgi apparatus; gc – gland cell; laz – lateroabfrontal zone; lfc – laterofrontal cell; lfz – laterofrontal zone; m – mouth; mc – muscle cell; mi – microvilli; mt – microtubule; n – nucleus; nf – nerve fiber; pp – remnants of the hood; pt – posterior part of the larval body with the telotroch; yt – youngest tentacle.

After 20 minutes, all catastrophic events of *P. harmeri* metamorphosis are completed. The preoral lobe remains as two dorsolateral hills, which contain an aggregation of perikarya (Figure 9A). Then, all changes inside the body occur, particularly those concerning the development of the nephromixium, the formation of blood vessels, and the reorganization of the coelomic system, including the appearance of the lateral mesenteries.

A 3-day-old juvenile looks very similar to an adult animal, but it still has a large posterior pouch, which contains the larval telotroch. In the 3-day-old juvenile, the reorganization of the tentacular apparatus finishes, the epistome is formed, and the remnants of the hood containing aggregations of perikarya are integrated into the juvenile epidermis (Figure 9B). Each tentacle has a definitive organization. The postoral ciliated band develops *de novo*, and new laterofrontal cells appear (Figure 9B, C). These cells are associated with a small laterofrontal neurite bundle that consists of 5 neurites (Figure 9D). The mediofrontal neurite bundle consists of 10–15 neurites and includes most basal neurites with dense core synaptic vesicles (Figure 9E). Several neurite bundles appear along the abfrontal side of each tentacle. The largest bundle consists of 20 neurites of different diameters (Figure 9F). The lateroabfrontal neurite bundle remains in the 3-day-old juvenile and is still associated with gland cells (Figure 9G). The tentacular neurite bundle consists of a huge aggregation of transversal neurites and perikarya of different types (Figure 9H). The aggregation of neurites forms the most basal layer, which contacts the basal lamina. Most of the neurites have a small diameter and an electron-dense cytoplasm filled with clear (electron-lucent) synaptic vesicles. Some neurites are large in diameter and have an electron-lucent cytoplasm and dense core synaptic vesicles (Figure 9H). The next layer is formed by nonsensory perikarya. The cytoplasm of these perikarya is filled with numerous rough endoplasmic reticula, mitochondria, and synaptic vesicles of different types. Some cells that contact the epidermal surface are most likely sensory because their basal portions are transformed into nerve projections. These cells bear microvilli, which surround a single cilium. An elongated nucleus bears the nucleolus and occupies the middle portion of the cell. The basal portion of the cell forms several projections, some of which contain microtubules and synaptic vesicles and pass through the epidermis to contact the basal lamina (Figure 9H).

**Figure 10:**
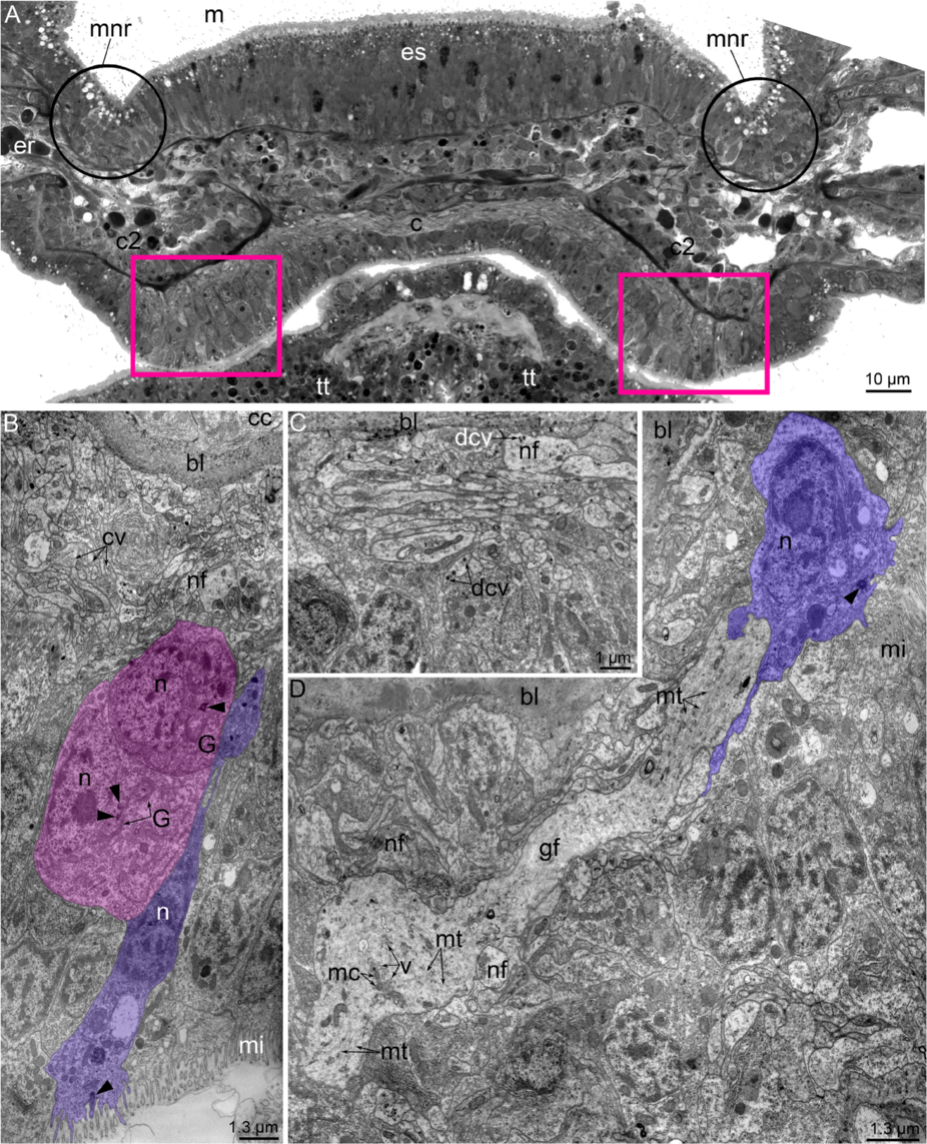
**Organization of the dorsal ganglion in 3-day-old juvenile of *Phoronopsis harmeri*.** Cross semithin **(A)** and thin **(B-D)** sections of the head region. **(A)** Two dorsolateral groups of perikarya (pink boxes) connect via thick commissure. **(B)** A portion of one of the dorsolateral group of perikarya: sensory (blue) and nonsensory (pink) perikarya are visible. Perikarya of both types bear centrioles (arrowheads). **(C)** A portion of the commissure, which consists of nerve fibers of different types. **(D)** A proximal portion of the giant nerve fiber associated with epidermal cell (blue), which contacts the epidermis surface and bears the centriole (arrowhead). Abbreviations: bl – basal lamina; c – commissure; c2 – mesocoel; cc – coelomic lining; cv – clear (electron-lucent) synaptic vesicle; dcv – dense-core vesicle; er – erythrocyte; es – esophagus; G – Golgi apparatus; gf – giant nerve fiber; m – mouth; mc – mitochondria; mi – microvilli; mnr – minor nerve ring; mt – microtubule; n – nucleus; nf – nerve fiber; tt – telotroch.

A 3-day-old juvenile contains an anlage of the dorsal ganglion. It consists of two dorsolateral groups of perikarya, which connect through a thick commissure (Figure 10A). Each dorsolateral group of perikarya includes different types of nerve cells. The perikarya of the first type are most likely sensory because they contact the epidermal surface and bear cilium and microvilli. The elongated nucleus has an electron-dense karyoplasm. The perikarya of the second type do not contact the epidermal surface and have a roundish nucleus with an electron-lucent karyoplasm (Figure 10B). Usually, the perikarya of the second type contain centrioles, which are associated with huge Golgi apparatuses. The aggregation of neurites is located between the perikarya of the second type and the basal lamina. The neurites are arranged in the transversal direction and form a large commissure between the two dorsolateral groups. As the tentacular nerve ring, the commissure consists of neurites of different types, which contain synaptic vesicles with electron-dense, electron-lucent, and electron-medium (with content of average electron dense) cores (Figure 10C). A 3-day-old juvenile has a giant nerve fiber, which included in the commissure, and can be recognized among other neurites due to its large diameter that can reach 4.5 μm (Figure 10D). The giant nerve fiber starts from the right dorsolateral group of perikarya and passes along the commissure to the left side of the body (Figure 10D). In the proximal portion, the giant nerve fiber is associated with epidermal cell with long thin projections that envelop the giant fiber. This envelope exists in the most proximal part of the giant nerve fiber, whereas it is absent distally where the giant nerve fiber contacts the other neurites of the commissure. The cytoplasm of the giant nerve fiber contains mitochondria that are small in diameter, electron-lucent synaptic vesicles, vacuoles, and microtubules that are oriented in different directions (Figure 10D).

**Figure 11:**
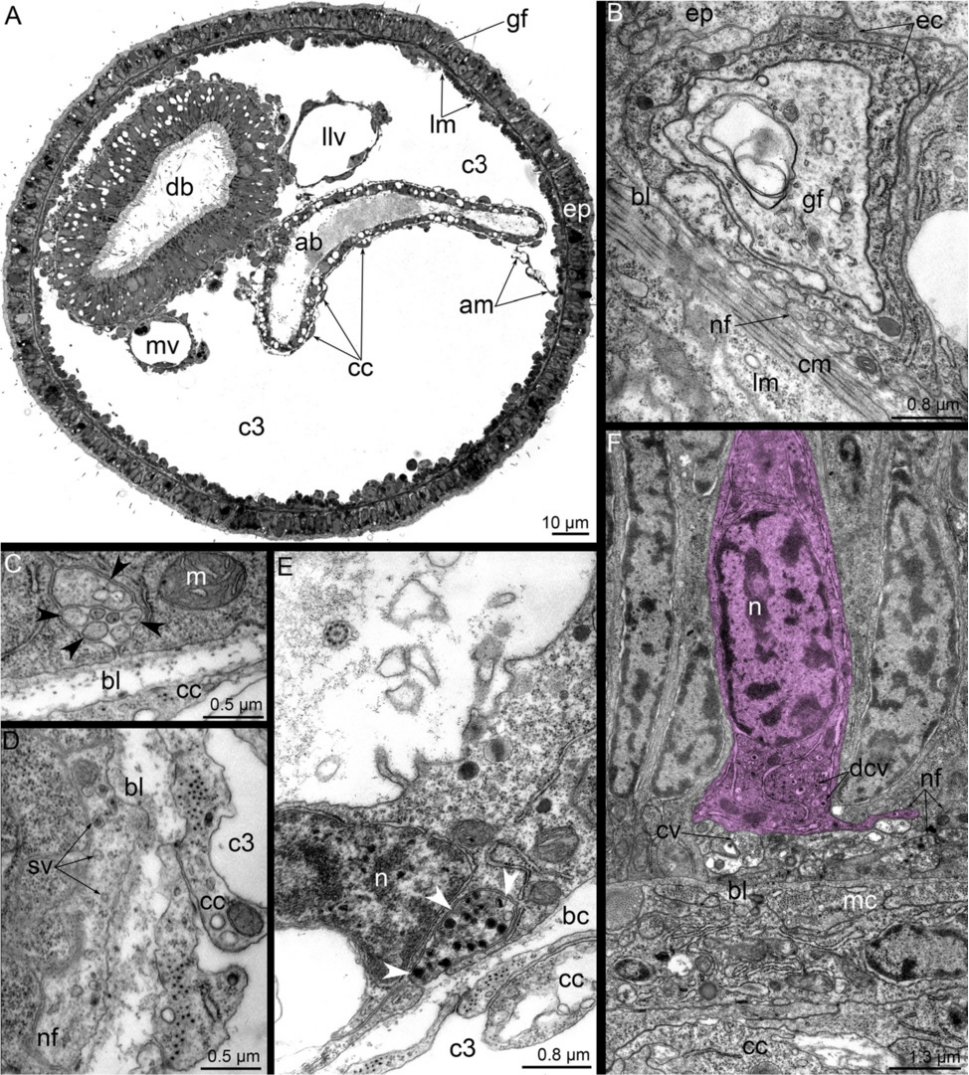
**Some nerve elements of the trunk in 3-day-old juvenile of *Phoronopsis harmeri.*** Cross semithin **(A)** and thin **(B-F)** sections of the anterior body part. **(A)** Whole section with formed definitive digestive tract and blood vessels. **(B)** The giant nerve fiber, which is completely enveloped by two cells and accompanied by several nerve fibers of common diameter. **(C)** Group of nerve fibers (arrowheads) in the epithelium of descending branch of the digestive tract. **(D)** Large projection of nerve cell contains synaptic vesicles and located in the epithelium of descending branch of the digestive tract. **(E)** Projection of neurosecretory cell (arrowheads) in the epithelium of ascending branch of the digestive tract. **(F)** Neuron (pink) with dense-core synaptic vesicles and nerve fibers, some of which contain clear (electron-lucent) vesicles in the epithelium of the esophagus. Abbreviations: ab – ascending branch of digestive tract; am – anal mesentery; bc – blastocoel; bl – basal lamina; c3 – trunk coelom; cc – coelomic lining; cv – clear (electron-lucent) synaptic vesicle; db – descending branch of digestive tract; dcv – dense-core vesicle; ec – enveloping cell; ep – epidermis; llv – left lateral blood vessel; lm – longitudinal muscles; m – mitochondria; mc – muscle cells; mv – median blood vessel; n – nucleus; nf – nerve fiber; sv – synaptic vesicles.

The giant nerve fiber is not very short in young juveniles, but it can be observed in the cross sections of the anterior part of the body (Figure 11A). Here, the giant nerve fiber has a diameter of approximately 2 μm, which is smaller than in the head region (Figure 11B). Projections of several cells form a complete envelope around the giant nerve fiber, and it does not contact the basal lamina or the neurites of the epidermal nerve plexus.

Nerve elements are found in different parts of the digestive tract, and these nerve elements are completely inherited from the larval stage. Perikarya and numerous neurites of different types are located in the epithelium of the esophagus (Figure 11F). Small neurite bundles, which consist of a few neurites, pass along the prestomach epithelium (Figure 11C). Here, the large projections of nerve cells, which is filled with synaptic vesicles, are located (Figure 11D). Sporadic neurites with dense core synaptic vesicles were found in the epithelium of the proctodaeum (the ascending branch of the digestive tract) (Figure 11E).

## Discussion

### Metamorphosis in phoronids

According to recent data [23], phoronid metamorphosis occurs in two different ways: with complete or incomplete reduction of organ systems. In *P. harmeri*[14], the metamorphic remodeling of the digestive tract, including the formation of the definitive proctodaeum and the fate of the larval telotroch, differs from that of *P. muelleri*[12] and looks very similar to that of *P. psammophila*[13]. Certain differences exist in the metamorphic remodeling of the muscles in *P. harmeri*[23] and *P. pallida*[15]. In *P. pallida*, all larval muscles undergo cell death, and definitive muscles form *de novo*[15]. However, in *P. harmeri*, the tentacle elevators, esophageal musculature, and trunk body musculature, including muscles of the blood vessels, are inherited by the juvenile from the larva and incorporated into a definitive muscular system [23].

The metamorphic remodeling of the larval nervous system is dissimilar in different phoronid species. This difference concerns the remodeling of tentacular neurite bundles. In *P. pallida*, all radial neurite bundles in the tentacles form *de novo*[15], whereas in *P. harmeri*, the lateroabfrontal neurite bundles, which contact the main nerve ring, are inherited from the larva. At the same time, in *P. harmeri*, the laterofrontal neurite bundles, which innervate the postoral ciliated band, form *de novo*, and their ultrastructure differs from that of the larval laterofrontal bundles (see [8]). The medioabfrontal neurite bundle is not present in larvae [8] but appears in juveniles. Thus, the juvenile has six radial neurite bundles in each tentacle: one mediofrontal, two laterofrontal, one medioabfrontal, and two lateroabfrontal (Figure 1A, B).

During metamorphosis in both *P. pallida* and *P. harmeri*, the apical organ undergoes complete destruction, and the main nerve ring is maintained [15], herein. A two-day-old juvenile of *P. pallida* lacks an anlage of the dorsal ganglion, and its final state is developed further post-metamorphosis [15]. In contrast, the juvenile of *P. harmeri* inherits two dorsolateral groups of perikarya, which give rise to the dorsal ganglion (Table 1). The presence of these groups of perikarya at the larval stage (Figure 1C) might be considered to be an embryonization of development and most likely correlates with the long life of *P. harmeri* larvae in plankton. Interestingly, the blood system, which is anatomically and histologically complex in adult phoronids [24,25], also forms in the larval stage [26].

The innervation of the inner organs is mostly inherited from the larva. Neurites and perikarya were found in all parts of the digestive tract in the larva, the metamorphic animal, and the juvenile.

In summary, the metamorphic remodeling of the phoronid nervous system occurs in two different ways. This difference concerns the fate of the larval tentacular neurite bundles and the formation of the definitive dorsal ganglion. The presence of different pathways of phoronid metamorphosis might correlate with a difference in phoronid biology [1] and the organization of the larval nervous system [27].

### Metamorphosis in phoronids and other Bilateria

During *P. harmeri* metamorphosis, some elements of the nervous system are lost, but others are integrated into the juvenile nervous system. The apical and frontal organs of the larva are completely consumed by the juvenile. This fate most likely reflects the provisional state of the apical and frontal organs as sensory structures, which is important for larval life in plankton and settlement [12].

A reduction of the apical organ is described in the larvae of both protostomes, including the nemerteans [28], caenogastropods [29], nudibranchs [30], and others [31], and deuterostomes [32,33]. The reduction occurs before or after metamorphosis and can exhibit cell death of the apical organ without catastrophic changes or with great remodeling of the body plan, when apical organ together with other body parts is completely consumed by the juvenile [28], herein.

Schmidt-Rhaesa [32] has expressed the view that, during metamorphosis, the apical organ has different fates in protostomes and deuterostomes. According to Schmidt-Rhaesa [32], in protostomes, the larval apical organ and adjacent nerve elements are usually integrated into the adult system completely or at least partially; whereas in deuterostomes, apical organ is completely destroyed during metamorphosis and does not incorporated into the definitive nervous system. For this reason, phoronids, whose apical organs do not integrate into the definitive nervous system, exhibit a “deuterostomian-like” type of metamorphosis. However, several elements of the larval nervous system are integrated into phoronid definitive nervous system, which eventually exists as combination of larval and definitive structures. The fusion of larval and definitive nerve elements has been established for deuterostomes and seems to have been inherited from the last common bilaterian ancestor [33].

### Innervation of the lophophore in “Lophophorata”

Among the three “Lophophorata” groups (Brachiopoda, Bryozoa, and Phoronida), the organization of the nervous system has been studied in greatest depth in adult phoronids [34-38]. All authors have described two main elements of the nervous system in adult phoronids: the nerve ring along the external row of tentacles (the main nerve ring), and the concentration of nerve cells and processes at the anal side, which is referred to as the ganglion [35-37,39,40].

According to previous studies, in phoronids, each adult tentacle contains either two groups of neurite bundles, frontal and abfrontal [38,41,42], or two laterofrontal neurite bundles, which connect the sensory cells of the tentacles and the nerve ring [35]. However, the efficient location of the neurite bundles in one tentacle has not been mentioned previously [41]. According to our results, the structure of the lophophore nervous system exhibits a regular alternation of intertentacular and abfrontal neurite bundles, which originate from the main nerve ring (Figure 1B, D). The prominent feature is the presence of intertentacular branches that give rise to the two neurite bundles that penetrate into adjacent tentacles.

The same intertentacular branches are known in adult bryozoans [43,44]. They originate from the circum-oral nerve ring that arises from the cerebral ganglion. The circum-oral nerve ring passes along the inner side of the lophophore base. Its position correlates with the location of the minor nerve ring in phoronids. In bryozoans, the intertentacular nerves branch into two pairs of neurites that penetrate into the adjacent tentacles. On the abfrontal side of the tentacle, one pair of neurites fuse and form the abfrontal nerve; the other pair forms the laterofrontal nerves. The frontal nerve independently originates from the main nerve ring of bryozoans [43]. In some bryozoans, the intertentacular nerves give rise to the thin lateral nerves, which contribute to the mediofrontal nerve [44]. The number and location of the radial tentacular neurite bundles in bryozoans is still uncertain [43].

Thus, the presence of intertentacular neurite bundles makes the innervation of the lophophore of phoronids appears similar to that of bryozoans. Moreover, according to some TEM data, both phoronids [36] and bryozoans [45] have not only subepidermal but also subperitoneal nerves. They pass along the lateral sides of each tentacle in bryozoans [45] and are irregularly scattered in phoronids [36]. On the other hand, innervation of the phoronid lophophore differs from that of bryozoans in the location of the main nerve ring and the origin and location of the radial tentacular neurite bundles. In bryozoans, the intertentacular branches give rise to the laterofrontal and abfrontal neurites bundles, whereas in phoronids, the intertentacular bundle gives rise to the lateroabfrontal tentacular neurite bundles. In contrast to phoronids, which have lateroabfrontal neurite bundles at both the larval [8] and adult (herein) stages, some bryozoans lack these bundles in tentacles [43]. The histological organization of the dorsal ganglion differs in phoronids and bryozoans. In bryozoans, the cerebral ganglion is invaginated into the epidermis [43,46], whereas in phoronids, the dorsal ganglion is located subepidermally. At the same time, the main nerve center is located on the dorsal side between mouth and anus in both phoronids and bryozoans.

Thus, we can conclude that the organization of the nervous system in phoronids and bryozoans differs, whereas many morphological similarities of the lophophore exist between the phoronids and bryozoans, including the locations of muscles and the presence of radial neurite bundles, different zones of the epidermis and specialized laterofrontal cells. The difference of lophophore innervation might reflect independent origins of the lophophore in phoronids and bryozoans that support molecular analysis data that suggests phoronids and bryozoans are not relatives at all.

Innervation of the brachiopod lophophore has not been clearly studied. According to some results [47,48], each brachium of the lophophore is innervated by lower and main brachial nerves, which arise from subenteric and supraenteric ganglions, respectively. These two ganglia are the main nerve elements of the brachiopod nervous system. Some brachiopods have accessory brachial nerves, which pass near the main brachial nerve. The supraenteric ganglion gives rise to several frontal neurite bundles, which pass along the frontal side of each tentacle. Interestingly, there are only frontal tentacular neurite bundles in brachiopods [48].

The innervation of the lophophore in brachiopods greatly differs in comparison with phoronids because of the difference in location and origin of the radial tentacular neurite bundles and the absence of intertentacular branches. On one hand, this difference might be due to poor study of the brachiopod nervous system. On the other hand, the difference might reflect a general difference between the body plan organization in phoronids and brachiopods. More detailed observations are needed on the organization of brachiopod nervous system to clarify the source of this difference.

### The organization of a definitive nervous system in phoronids and other bilaterians

The organization of the phoronid definitive nervous system is traditionally regarded as one of the most primitive types of organization among all bilaterians [32,38,49]. This opinion is based on the peculiarities of histological organization of the phoronid definitive nervous system. It is organized as a subepidermal nerve plexus, which is completely located in the epidermis, and can be compared with the nerve plexus of cnidarians [40]. Among all Bilateria, the definitive nervous system, which is completely located in the epidermis, can be found in both protostomes and deuterostomes, including vestimentiferans [50], hemichordates [51], and echinoderms [52]. However, the definitive nervous system of phoronids is unique compared with these animals because it lacks longitudinal nerves.

Although the nervous system of phoronids is organized as a nerve plexus, it exhibits centralization at an early stage of formation of the definitive nervous system. This fact supports the hypothesis that a nerve center was present in the last common bilaterian ancestor [53,54].

The dorsal ganglion of juvenile phoronids consists of two dorsolateral groups of perikarya, which connect through a thick commissure. The serotonin-like immunoreactive dorsal commissure between two branches of the main nerve ring was found in early larvae of *P. harmeri*[9]. In competent larvae, this dorsal commissure does not exhibit immunoreactivity against serotonin [8]. This type of organization of the nerve center, which consists of two groups of perikarya connected through a commissure, is regarded as a “commissural brain” [31]. Other bilaterians, including the lower bilaterian Acoelomorpha, have been reported to have a similar commissural brain [31,55]. Because Acoelomorpha has been recently established as a deuterostome bilaterian [56], the “commissural brain” is now present in all three large stems of Bilateria: Deuterostomia (Acoelomorpha), Ecdysozoa (Arthropoda), and Lophotrochozoa (Gastrotricha, Phoronida). This dispersion might reflect the presence of the “commissural brain” in the last common bilaterian ancestor.

### A scenario of phoronid evolution

**Figure 12:**
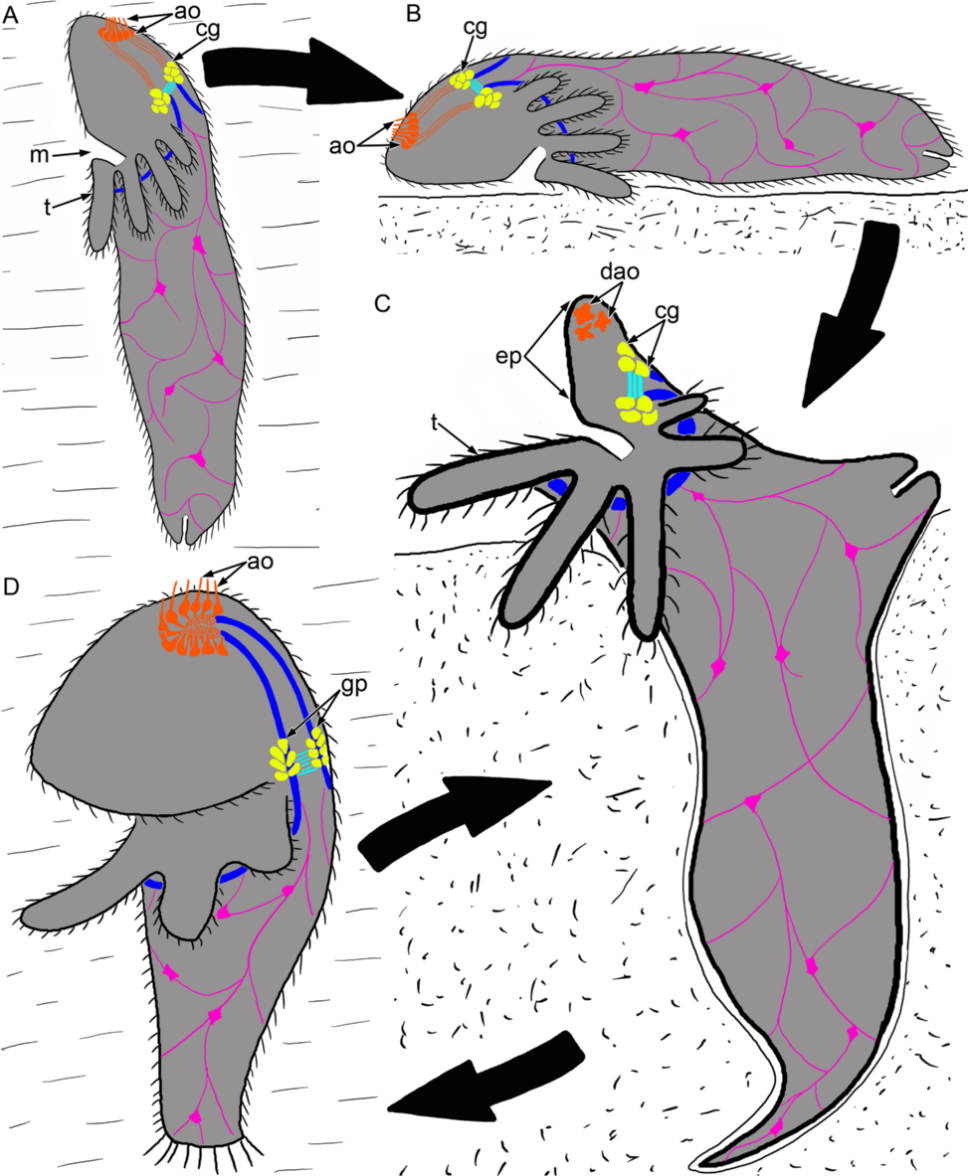
**Scenario of phoronid body plan evolution.** Different elements of the nervous system are indicated by different color: orange – apical organ, yellow – cerebral ganglion; cyan – the commissure, blue – nerve tract, which innervates the tentacles, pink – neurites and perikarya of the nerve plexus. **(A)** Hypothetical pelagic ancestor with tentacles and ciliated epidermis. **(B)** Creeping ancestor. **(C)** The formation of ventral pouch, which is exploited as a tool to dig into soft substrata to avoid danger. The apical organ undergoes the reduction. **(D)** Appearance of actinotroch larva. Abbreviations: ao – apical organ; cg – cerebral ganglion; dao – degenerating apical organ; ep – epistome; gp – dorsolateral groups of perikarya; m – mouth; t – tentacle.

The presence of the apical organ at the larval stage in many bilaterians and its reduction prior to or during metamorphosis allow to suggest that the apical organ is associated with the pelagic part of life cycles [57]. This provides evidence in support of the Trochaea theory [58], according to which the last common bilaterian ancestor was a pelagic form with the apical organ and cerebral ganglia. This hypothesis also gains support in our study, where we report the co-existence of both the apical organ and anlage of the cerebral ganglia in phoronid larvae. These observations suggest the presence of the apical organ in the phoronid ancestor – a pelagic animal possessing the cerebral ganglia that connect the neurite bundle, which innervates the row of tentacles (Figure 12A). This pelagic form adopted a crawling life style (Figure 12B), which triggered dramatic changes in its morphology associated with the formation of an active ventral pouch (Figure 12C). This pouch was exploited as a tool to dig into soft substrata to avoid danger. The apical organ reduced as no longer functional in sessile life. The actinotroch larva had evolved as a dispersion stage (Figure 12D). It possessed the apical organ and used catastrophic metamorphosis to mature into adult, which now recapitulates the ancient evolutionary innovation of phoronids.

## Conclusions

The metamorphic remodeling of the phoronid nervous system occurs in different ways. The definitive nervous system combines larval and adult nerve elements. The same combination is known in deuterostomes and was inherent in the last common bilaterian ancestor. The nervous system of the juvenile (and the adult) is organized as an epidermal plexus but demonstrates a concentration in the anterior portion of the body. The presence of the concentration of perikarya and neurites in the anterior portion of the body – a nerve center that forms a “commissural brain” – is characteristic of the last common bilaterian ancestor. The dorsal ganglion of phoronids also forms a “commissural brain”. Thus, phoronids demonstrate some plesiomorphic features that were inherited from the last common bilaterian ancestor and maintained through time. Because phoronids exhibit certain plesiomorphic features, they can be regarded as one of the most primitive groups of lophotrochozoans.

## Methods

### Animals

Metamorphic animals and newly formed juvenile of *P. harmeri* were collected with a planktonic net during November of 2011 in Vostok Bay, Sea of Japan. Larvae were reared at 1 to 3C in an incubator with a 12-h light–dark cycle until metamorphosis and then until 3- and 10-day-old juveniles. At 2–3 min intervals (up to the newly formed juvenile), specimens were prepared for future investigations (see below).

### Light microscopy

Metamorphic stages, newly formed juveniles, and 3- and 10-day-old juveniles were photographed using a Panasonic DMC-TZ10 digital camera mounted on a binocular light microscope. All these stages were prepared for scanning electron microscopy (SEM), transmission electron microscopy (TEM), cytochemistry, and confocal laserscanning microscopy (CLSM).

### Electron microscopy

For SEM, fixed metamorphic stages of *P. harmeri* that had been dehydrated in ethanol followed by an acetone series were critical point dried and then sputter coated with platinum-palladium alloy. Specimens were examined with a Jeol JSM scanning electron microscope.

For TEM, metamorphic stages and 3-day-old juveniles of *P. harmeri* were fixed at 4˚C in 2.5% glutaraldehyde in 0.05 M cacodylate buffer containing 21 mg/ml NaCl and then postfixed in 2% osmium tetroxide in the same buffer containing 23 mg/ml NaCl. Postfixation was followed by *en bloc* staining for 2 h in a 1% solution of uranyl acetate in distilled water. Specimens were then dehydrated in ethanol followed by an acetone series and embedded in Spurr resin (Sigma Aldrich). Semithin and thin sections were cut with a Reichert Ultracut E ultratome. Semithin sections were stained with methylene blue, observed with Zeiss Axioplan2 microscope and photographed with an AxioCam HRm camera. Thin sections were stained with lead citrate and then examined with a JEOL JEM 100B electron microscope.

### Cytochemistry

For cytochemistry, metamorphic stages of *P. harmeri*, newly formed juveniles, 3-, and 10-day-old juveniles were narcotised in MgCl2, then fixed overnight in a 4% paraformaldehyde solution on a filtrate of sea water and washed (two times) in phosphatic buffer (pH 7.4) (Fisher Scientific) with Triton X-100 (0.3%) (Fisher Scientific, Pittsburgh, PA, USA) for a total of 2 h. Nonspecific binding sites were blocked with 1% normal donkey serum (Jackson ImmunoResearch, Newmarket, Suffolk, UK) in PBT overnight at +4°C. Subsequently, the larvae were transferred into primary antibody: the mixture of a-Acetylated Tubulin (1:1000) and either anti-FMRFamide (1:3000) or anti-serotonin (1:1000) (ImmunoStar, Hudson, WI, USA) in PBT and incubated for 24 h at +4°C with gentle rotation. Specimens were washed for 8 h at +4°C (at least three times) in PBT and then exposed to the secondary antibody: donkey anti-rabbit- Atto 647 N and donkey anti-mouse-Atto 565 (Invitrogen, Grand Island, NY, USA) both 2–3 mkg/ml in PBT for 24 h at +4°C with gentle rotation. Then, the specimens were washed in PBT/BSA and incubated in a mixture of rhodamine-conjugated phalloidin (1:50) (Fisher Scientific, Pittsburgh, PA, USA). In the following, they were washed in PBS (three times × 40 min), mounted on a cover glass covered with poly-L-lysine (Sigma-Aldrich, St. Louis, MO, USA), and embedded in Murray Clear. Specimens were viewed with Leica TCS SP5 confocal microscope (IDB, Moscow, Russia). Z-projections were generated using the programme Image J version 1.43.

### Ethical approval

The use of phoronids in the laboratory does not raise any ethical issues and therefore approval from regional or local research ethics committees is not required.

## Competing interests

The authors declare that they have no competing interests.

## Authors’ contributions

ENT designed and coordinated research, performed research including some staining and confocal research, TEM investigations, analyzed data, prepared all figures, and wrote the manuscript. EBT performed staining and confocal research. All authors conceived the study, read, and approved the final version of the manuscript.

## Supplementary Material

Additional file 1: Movie 1 The start of metamorphosis in *Phoronopsis harmeri*. This time-lapse movie shows the typical first steps of the metamorphosis: eversion of the metasomal sac, strong contractions of the ampulla and anterior portion of the body, and maceration of the hood. The active movement of coelomic fluid induces the break of larval blood masses and flow of erythrocytes into the blood vessels. Click here for file
